# Carriage of healthcare-associated methicillin-resistant *Stapyloccous aureus* and empiric treatment for skin and soft tissue infections

**DOI:** 10.1186/1753-6561-5-S6-P17

**Published:** 2011-06-29

**Authors:** I Uckay, A Reber, A Moldovan, N Dunkel, S Emonet, A Agostinho, G Renzi, J Schrenzel, S Harbarth

**Affiliations:** 1 Surgery. Internal Medicine/Infection Control Program, Geneva University Hospitals, Geneva, Switzerland; 2 Internal Medicine, Geneva University Hospitals, Geneva, Switzerland; 3 Direction médicale/Infection Control Program, Geneva University Hospitals, Geneva, Switzerland; 4 Laboratory of Bacteriology, Geneva University Hospitals, Geneva, Switzerland

## Introduction / objectives

Previous skin carriage of healthcare-associated methicillin-resistant *Staphylococcus aureus* (HA-MRSA) leads frequently to empiric MRSA coverage for the antibiotic treatment of skin and soft tissue infections.

## Methods

Retrospective cohort study between January 1996 and -June 2010 including adult orthopedic patients hospitalized at Geneva University Hospitals (MRSA prevalence; 30%).

## Results

A total of 378 skin and soft tissue infections in 346 patients were retrieved. Among all episodes, 102 revealed a positive current MRSA status (during 2 weeks preceding infection; 102/378; 27%) and 70 (19%) were MRSA carriers in the past. The sensitivity, specificity, positive and negative predictive values of current MRSA skin carriage to predict abscesses due to MRSA were 0.68, 0.77, 0.19, and 0.97, respectively. Fifty-four current MRSA carriers (54/102, 53%) and 30 past carriers (43%) were successfully treated with a non-MRSA antibiotic agent. In multivariate Cox regression analysis, anti-MRSA antibiotic coverage (hazard ratio 1.2, 95% CI 0.5-2.8) and duration of antibiotic therapy (HR 1.0, 95% CI 0.96-1.02) did not influence treatment failure among patients with positive MRSA carriage, in contrast to presence of immune suppression (HR 7.8, 95% CI 1.8-34.1).

## Conclusion

Current or past HA-MRSA skin carriage poorly predicts the need for anti-MRSA coverage for the antibiotic treatment of skin and soft tissue infections in hospitalized orthopaedic patients.

## Disclosure of interest

None declared.

